# Return-to-fly after flow diversion for unruptured paraclinoid aneurysms in military pilots

**DOI:** 10.3389/fneur.2026.1709319

**Published:** 2026-01-14

**Authors:** Hui Zhang, Cheng-Ye Zhang, Cheng-Cheng Zhao, Yan Zhou, Yu-Dong Ma

**Affiliations:** 1 Department of Neurosurgery, Air Force Medical Center of the PLA, Beijing, China; 2 Department of Medical Identification, Air Force Medical Center of the PLA, Beijing, China

**Keywords:** aviation medical certification, flow diversion, military pilot, neurointervention, paraclinoid aneurysms

## Abstract

**Objective:**

Advances in neuroimaging have increased detection of unruptured paraclinoid aneurysms among military pilots, a population subjected to unique environmental stressors that may elevate rupture risk. However, optimal management remains clinically uncertain. This study introduced the procedural safety, occlusion efficacy, and flight requalification outcomes following flow diversion (FD) treatment in this cohort.

**Methods:**

A retrospective review was conducted on military pilots who underwent FD treatment for unruptured paraclinoid aneurysms between 2019 and 2024. Demographic, aneurysm, and treatment data were collected. Outcomes included procedural success, occlusion status (assessed by the Modified O’Kelly-Marotta (OKM) scale), and flight requalification status.

**Results:**

Four pilots with five aneurysms underwent successful FD implantation. All achieved complete aneurysm occlusion (OKM-D) at 12-month follow-up without thromboembolic or hemorrhagic complications. Two high-performance fighter pilots initially received dual-seat restrictions before full clearance; one helicopter pilot and one transport aircraft pilot were cleared directly. All pilots successfully returned to flight duties, accumulating 80–200 safe flight hours post-treatment.

**Conclusion:**

FD treatment for unruptured paraclinoid aneurysms in military pilots is safe and effective, facilitating timely return to flight duty with appropriate aeromedical evaluation.

## Introduction

Enhanced cerebral magnetic resonance imaging (MRI) technology has increased detection of incidental unruptured intracranial aneurysms (UIAs) among young pilot candidates and active pilots (1). This population faces unique environmental stressors—hypergravity and hypobaric hypoxia—that disrupt cerebral hemodynamics and potentially elevate aneurysm rupture risk (2–5). Paraclinoid aneurysms is a distinct clinical subtype which originates from the internal carotid artery (ICA) between the distal dural ring and the posterior communicating artery’s origin, encompassing the clinoidal and ophthalmic segments (6, 7). In the 35–75 age group in China, the paraclinoid ICA harbors the majority (>50%) of UIAs (8). Optimal management strategies for unruptured paraclinoid aneurysms in this population remain clinically uncertain due to insufficient evidence-based guidance.

For active pilots, clipping is generally not recommended due to its prolonged recovery period and higher seizure risk (9, 10). Furthermore, from an aerospace medicine perspective, concerns exist regarding potential displacement of externally placed subarachnoid clips under sustained high +Gz forces due to physical inertia (11–14). Consequently, minimally invasive endovascular treatments, offering faster recovery and reduced operational interruption, are preferred to facilitate timely return to flight duties (15, 16).

Flow diversion (FD) promotes aneurysm occlusion by diverting flow away from the sac, reducing intra-aneurysmal shear stress, and inducing thrombosis (17). Compared to clipping, FD treatment offers a minimally invasive intervention with high technical success rates (18). Notably, no peer-reviewed studies or clinical registries have specifically reported FD utilization for unruptured paraclinoid aneurysms management in active-duty military pilots globally. This retrospective study analyzes FD-treated unruptured paraclinoid aneurysm cases in active-duty military pilots at our center. We evaluate procedural safety, occlusion efficacy, and aeromedical requalification outcomes to establish FD’s role for expediting return to flight status.

## Methods

### Study design

We systematically reviewed military pilots diagnosed with unruptured paraclinoid aneurysms undergoing FD treatment between 2019 and 2024. Data extraction encompassed demographic parameters (age, aircraft type, cumulative flight time), aneurysm characteristics (size, location), therapeutic outcomes (Modified O’Kelly-Marotta (OKM) scale at 12-month follow-up) and aeromedical disposition. The study protocol was approved in advance by the ethics committee. The requirement for informed consent was waived.

### Inclusion and exclusion criteria

The inclusion criteria for participants were as follows: (1) patients hospitalized in our center from January 2019 to December 2024; (2) active military pilots of the PLA Navy, Army, and Air Force; (3) clinically diagnosed unruptured paraclinoid aneurysms patients who received FD treatment. All inclusion criteria should be met.

The exclusion criteria were as follows: (1) non-active military flight personnel; (2) ground crews (air traffic controllers, photographers, flight attendants, etc.); (3) patients who refuse to fly or fail to complete aviation medical evaluation. Patients with any of the above conditions were excluded.

### Flight qualification assessment

Flight readiness must be assessed by the specialists at the Aerospace Medical Association of Air Force Medical Center. No pilot was granted the waiver unless the specialists initially recommended clearance to return to flight status. Flight requalification requires: (1) complete aneurysm occlusion confirmed by digital subtraction angiography (DSA), (2) absence of clinically significant in-stent stenosis or delayed neurovascular complications (3) cessation of antiplatelet therapy.

## Results

### Clinical features

Between January 2019 and December 2024, a total of 15 military active pilots were diagnosed with unruptured paraclinoid aneurysms, which accounted for the majority of UIAs (15/22 patients) at our center (19). Following comprehensive evaluation, which prioritized aneurysm size (>5 mm), morphology, multiplicity, and patient-specific factors, 11 cases with typically smaller, regular aneurysms without high-risk features were managed conservatively. The remaining 4 cases, including two with smaller aneurysms (2 mm and 3 mm) treated due to higher-risk morphology or bilateral presentation, underwent FD implantation and formed the research cohort (see Table 1). All patients were male and asymptomatic, and the mean age at diagnosis was 39 years (range, 31–45 years). Five unruptured paraclinoid aneurysms were detected in four patients, including one case of bilateral paraclinoid aneurysms. By aircraft type, one flew a helicopter, one flew a transport aircraft, and two flew high-performance fighter aircraft. Mean flying time was 2,875 h (range, 1,000–6,000 h).

**Table 1:** Key characteristics of four military pilots who underwent FD implantation.

Patient no.	Age (years)	Aircraft type	Flight hour (h)	Size of UIA (mm)	Location of UIA	FD device	Follow-up DSA assessment	Aeromedical assessment	Post-op flight hour (h)
1	44	Fighter aircraft (high performance)	2,300	4.2 mm × 2.1 mm	Paraclinoid aneurysm (L)	Pipeline™ Flex Embolization Device (Medtronic, USA)	OKM grade D	Dual-seat first, cleared at 6mo	200
2	36	Fighter aircraft (high performance)	2,200	3.0 mm × 2.5 mm	Paraclinoid aneurysm (L); Superior	Pipeline™ Flex Embolization Device (Medtronic, USA)	OKM grade D	Dual-seat first, cleared at 6mo	180
3	45	Transporter	6,000	5.1 mm × 4.9 mm	Paraclinoid aneurysm (R)	Pipeline™ Flex Embolization Device (Medtronic, USA)	OKM grade D	Full clearance	150
4	31	Helicopter	1,000	8.9 mm × 5.4 mm (R), 2.0 mm × 1.2 mm (L)	Paraclinoid aneurysm (bilateral)	Pipeline™ Flex Embolization Device (Medtronic, USA)	OKM grade D	Full clearance	80

DSA, digital subtraction angiography; FD, flow diverter; ICA, internal carotid artery; L, left; OKM, modified O’Kelly-Marotta scale; R, right; UIA, unruptured intracranial aneurysm.

All patients underwent successful FD implantation and were placed on a postoperative antiplatelet regimen for a total of 15 months. Specifically, they received dual-antiplatelet therapy (DAPT) for the initial 3 months, which was then stepped down to single-antiplatelet therapy (SAPT) for the subsequent 12 months. This protocol aligns with the recommendations of the Chinese guideline for the management of intracranial aneurysms with FD (20). Follow-up DSA at 12 months postoperatively demonstrated complete aneurysm occlusion (OKM scale D) in all patients without in-stent stenosis.

### Special issuance authorization

Pilots underwent special medical flight certification after complete intracranial aneurysm occlusion and discontinuation of antiplatelet therapy. One transport aircraft pilot and one helicopter pilot were granted full flight clearance, while two high-performance fighter pilots initially received restricted clearance limited to dual-seat operations. After 6 months of restricted operations, both pilots demonstrated asymptomatic performance during the evaluation period, resulting in removal of all flight restrictions with full clearance granted. All pilots subsequently logged an average of 153 safe flight hours (range, 80–200 h) without reporting any adverse physical reactions.

### Illustrative case

#### Case 1

**Figure 1:**
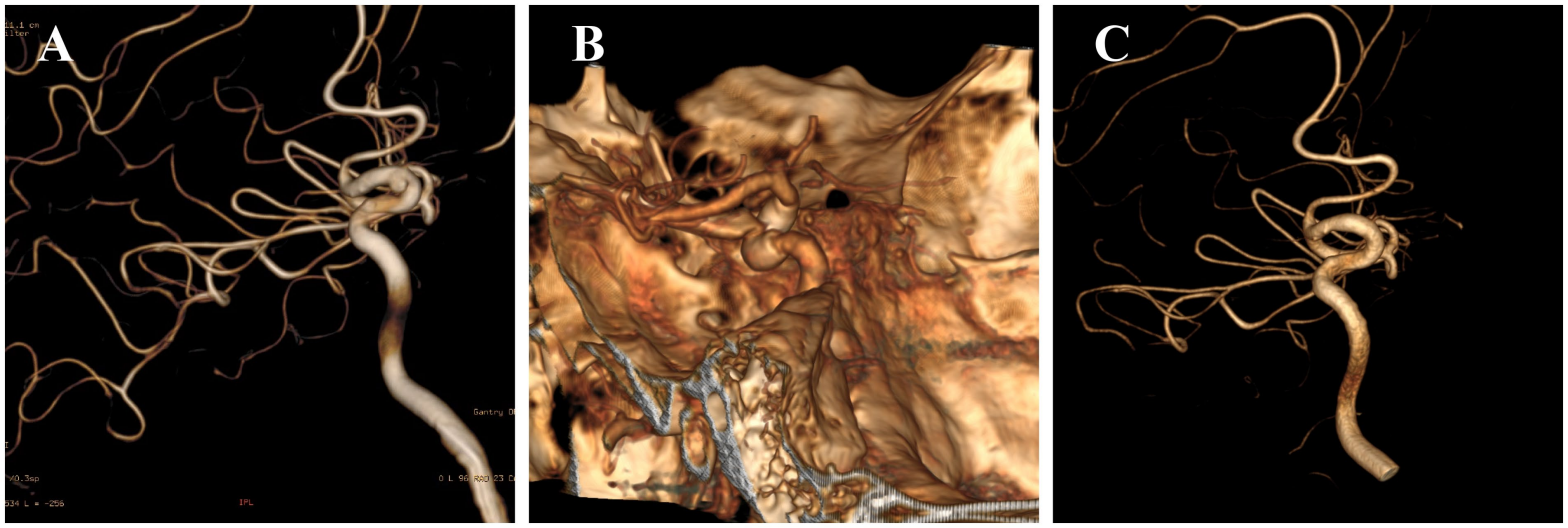
**(A)** A 44-year-old male high-performance jet fighter pilot (case 1) was diagnosed with a left paraclinoid internal carotid artery aneurysm. **(B,C)** Follow-up DSA at 12 months postoperatively demonstrated complete aneurysm occlusion (OKM scale D) in all patients without in-stent stenosis.

A 44 years old male, a high-performance jet fighter pilot, was found to have a left ICA aneurysm in the paraclinoidal segment during a physical examination in September 2021 (Figure 1A). It was approximately 4.2 mm × 2.1 mm and was asymptomatic, with no neurological dysfunction. The patient underwent FD implantation in October 2021, and was given a standard 15 months course of antiplatelet therapy after surgery. Follow-up DSA in October 2022 indicated complete aneurysm occlusion, graded as OKM D, without in-stent stenosis (Figures 1B,C). The patient was cleared for flight duty with restrictions limited to dual-seat operations. Then, the pilot’s flight restriction to dual-seat operations was lifted after 6 months of asymptomatic performance. Postoperatively, the patient safely completed 200 flight hours without any adverse symptoms.

## Discussion

Unruptured paraclinoid aneurysms in military pilots can rupture suddenly during missions, leading to subarachnoid hemorrhage (SAH) with severe headache, vomiting, confusion, or mental issues. This directly risks flight safety and mission success, requiring aggressive therapeutic interventions.

### Advantages of FD in treating unruptured paraclinoid aneurysms

The FDs represent a transformative advancement in endovascular UIA management over the past two decades, shifting the therapeutic paradigm from sac packing to durable vessel reconstruction via intraluminal scaffolding (18). By promoting hemodynamic modification rather than direct aneurysm occlusion, these devices enable treatment of complex aneurysms previously refractory to conventional endovascular techniques (21). Anatomically, the specific localization of aneurysms in our cohort—exclusively within the paraclinoid ICA segment—further underscores the clinical value of FD therapy. The complex regional anatomy (anterior clinoid process, cavernous sinus, optic apparatus) renders microsurgical clipping particularly challenging, solidifying endovascular management as the preferred approach. Contemporary evidence strongly supports FD superiority for paraclinoid ICA aneurysms. Di Maria et al. (22) demonstrated significantly better long-term occlusion with FD versus stent-assisted coiling (SAC). Burrows et al. (23) reported a 96% complete occlusion rate with no permanent adverse visual outcomes, while Zanaty et al. (24) achieved 77% complete occlusion and a remarkably low complication rate (2.3%). Long-term outcomes from Levy et al. (25) (median follow-up: 44.9 months; *n* = 113) revealed complete occlusion (Raymond-Roy class I) in 84.2% of cases. A recent meta-analysis corroborates these findings, indicating FD may yield higher complete occlusion rates at follow-up compared to SAC for paraclinoid aneurysms (6). Consequently, in military pilots with unruptured paraclinoid aneurysms, FD treatment offers a higher aneurysm occlusion rate while minimizing the risk of long-term complications, which is critical for ensuring their long-term health and occupational safety.

### Risks of FD in treating unruptured paraclinoid aneurysms

Compared to conventional stents, FDs exhibit higher metal coverage and denser stent mesh, which induces hemodynamic flow diversion, promotes intra-aneurysmal thrombosis, and facilitates neointimal formation across the aneurysm neck (18). Additionally, immediate embolization rate was low after FD surgery (18). Therefore, for military pilots, the main risks of FD in treating UIA are neurological dysfunction caused by ischemia and delayed hemorrhage.

#### Thromboembolic complications

Thromboembolic complications represent a key consideration in FD therapy. The devices’ high metal coverage significantly alters intra-stent hemodynamics, promoting thrombus formation that risks acute stent or parent artery occlusion, potentially leading to peri-procedural stroke (26). The paraclinoid location of all aneurysms in this cohort heightens concern for ophthalmic complications. Both surgical clipping and coiling carry inherent risks of ophthalmic artery occlusion and visual impairment, which is potentially career-ending for pilots. SAC for paraclinoid aneurysms demonstrates complication rates ranging from 1.4 to 15.4% (27–30). In contrast, FD therapy offers superior safety for visual outcomes. Touzé et al.’s (31) meta-analysis found FD achieved 85% occlusion with new visual symptoms in only 3.0% of cases. Silva et al. (32) demonstrated FD yielded the highest rate of visual improvement (71% vs. 58% clipping, 49% coiling) and the lowest rate of visual deterioration (5% vs. 11% clipping, 9% coiling) among patients with pre-existing visual symptoms. New visual deficits were found in patients with intact baseline vision at a rate of 1% for clipping, 0% for coiling, and 0% for FD. A recent meta-analysis by Tang et al. (6) found no difference in new visual impairment but revealed a higher complete occlusion rate after FD treatment compared with coiling. Moreover, some studies using optical coherence tomography angiography have demonstrated that FD coverage of the ophthalmic artery origin may reduce blood flow, but the actual incidence of clinical symptoms remains low due to the rich anastomotic network in the ophthalmic circulation (33). This compelling evidence positions FD as a safe therapeutic option for preserving visual function in pilots with unruptured paraclinoid aneurysms.

#### Hemorrhagic complications

Hemorrhagic complications represent another critical consideration. While redirecting flow away from the aneurysm, FDs simultaneously modify distal parent artery hemodynamics, potentially increasing risks of delayed aneurysm rupture or intraparenchymal hemorrhage (34). This risk peaks early, with approximately 75% of hemorrhages occurring within 30 days post-procedure (35). Brinjikji et al. (36) reported intracerebral hemorrhage in 2.5% of 906 aneurysms treated with Pipeline Embolization Device (PED), identifying ruptured aneurysm status and deployment of ≥3 devices as significant risk factors. Crucially, hemorrhagic events in unruptured paraclinoid aneurysms treatment remain relatively uncommon. Consequently, military pilots who have successfully surpassed the early high-risk period post-FD implantation face a remote probability of bleeding-related flight incapacitation.

Notably, DAPT is widely regarded as essential for preventing thromboembolic complications after FD implantation. However, prolonged platelet inhibition inevitably carries a risk of bleeding. Major bleeding events—defined as fatal bleeding, symptomatic bleeding into a critical organ (e.g., hemorrhagic stroke), bleeding causing a hemoglobin drop ≥2 g/dL, or bleeding requiring transfusion of ≥2 units of blood—remain relatively uncommon, with reported incidences generally below 1% (37, 38). Recent meta-analyses suggest that short-term DAPT (≤6 months) provides comparable protection against ischemic events while significantly reducing the risk of major bleeding compared to long-term DAPT (>6 months) (39). In our cohort, all four pilots received a standardized antiplatelet protocol consisting of 3 months of DAPT followed by 12 months of SAPT, with no ischemic or hemorrhagic complications observed. This supports the safety and efficacy of this regimen in this special population.

#### Stent migration

Delayed FD migration represents a critical long-term safety consideration despite high occlusion rates. Progressive displacement may occur post-implantation, potentially re-exposing the aneurysm to hemodynamic stress and risking vascular rupture, obstruction, or thromboembolic events—any of which could compromise pilot operational capacity (18). Tsai et al. (40) observed spontaneous delayed displacement in 4.9% of cases, with literature suggesting incidence rates of 2.2–4.9% and associated mortality reaching 40% in reported cases. Displacement timing varies widely (3 days to 14 months postoperatively). Key risk factors include stent-vessel size mismatch, suboptimal apposition, vessel tortuosity, thrombus-mediated drag forces, and—most critically—incomplete endothelialization of the high-metal-density device (40). Animal models indicate complete endothelialization requires weeks to months, necessitating extended surveillance (41). In this cohort, all four pilots underwent >12 months of post-implantation monitoring to mitigate this risk and ensure flight safety.

### Aviation medical certification

In this study, four pilots with unruptured paraclinoid aneurysms successfully underwent FD implantation without perioperative complications (ischemic/hemorrhagic events or neurological deficits). Follow-up angiography at 12 months confirmed complete aneurysm occlusion with preserved parent artery patency, and complete stent endothelialization was achieved, thereby eliminating concerns regarding in-flight device displacement and subsequent pilot incapacitation. Following aeromedical evaluation, two pilots (one transport aircraft, one helicopter) were cleared for unrestricted flight duty. The two high-performance fighter pilots, however, were initially restricted to dual-seat operations. This precautionary measure was implemented due to the substantially higher +Gz exposure and associated hemodynamic stresses inherent in fighter aviation (4, 5), the unknown long-term impact of such forces on the endothelialized flow diverter, and the need for a phased re-adaptation after a prolonged flight hiatus (>15 months). After 6 months of incident-free dual-seat operations, demonstrating both device stability and pilot readaptation, all restrictions were lifted. All four pilots subsequently resumed full flight duties without reporting any symptoms or performance limitations during follow-up.

### Study limitations

This study has several limitations. First, the small sample size and relatively short follow-up period may not fully represent the safety and efficacy profile of FDs; thus, we cannot conclude that FD is the optimal treatment approach. Second, there is currently no clinical or experimental data addressing the potential risk of FD migration under flight conditions, particularly during sustained +Gz acceleration, although animal studies have confirmed that inferior vena cava filters remain unaffected by high gravitational acceleration (42). Accordingly, we will continue to follow up on our cases. Third, although some studies have indicated an association between alterations in hemodynamic stress on the vessel wall and the observed in-stent stenosis, it remains unknown whether acute hemodynamic changes during flight could contribute to in-stent stenosis (43, 44). Lastly, our treatment outcomes for paraclinoid aneurysms might not be applicable to aneurysms in all locations. These unanswered questions highlight the need for longer-term follow-up studies with larger cohorts to further validate the safety of FDs in aviators.

## Conclusion

This study represents the first real-world investigation of aeromedical certification in pilots with intracranial aneurysms treated with FDs. The findings suggest that FD treatment for unruptured paraclinoid aneurysms in active-duty military pilots appears to be a safe and effective intervention, which may facilitate a timely return to flight duties. In this small cohort, all four treated pilots achieved complete aneurysm occlusion (OKM grade D) without experiencing thromboembolic or hemorrhagic complications, and all were successfully recertified for flight operations. The paraclinoid internal carotid artery location, a challenging anatomical region, supports the consideration of FD therapy as a favorable option for this specific population, as it balances high occlusion rates with relatively low procedural risks. These results provide preliminary evidence to inform the decision-making of aviation medical authorities regarding the certification of FD-treated pilots. Further studies with larger cohorts and longer follow-up are warranted to confirm these findings.

## Data Availability

The original contributions presented in the study are included in the article/supplementary material, further inquiries can be directed to the corresponding author.
